# Supplementary figures and images for: Tumor-Associated Protein Profiles in Kaposi Sarcoma and Mimicking Vascular Tumors, and Their Pathological Implications
Source: Int J Mol Sci. 2019 Jun 27;20(13):3142. doi: 10.3390/ijms20133142 (PMC6651042; doi:10.3390/ijms20133142)

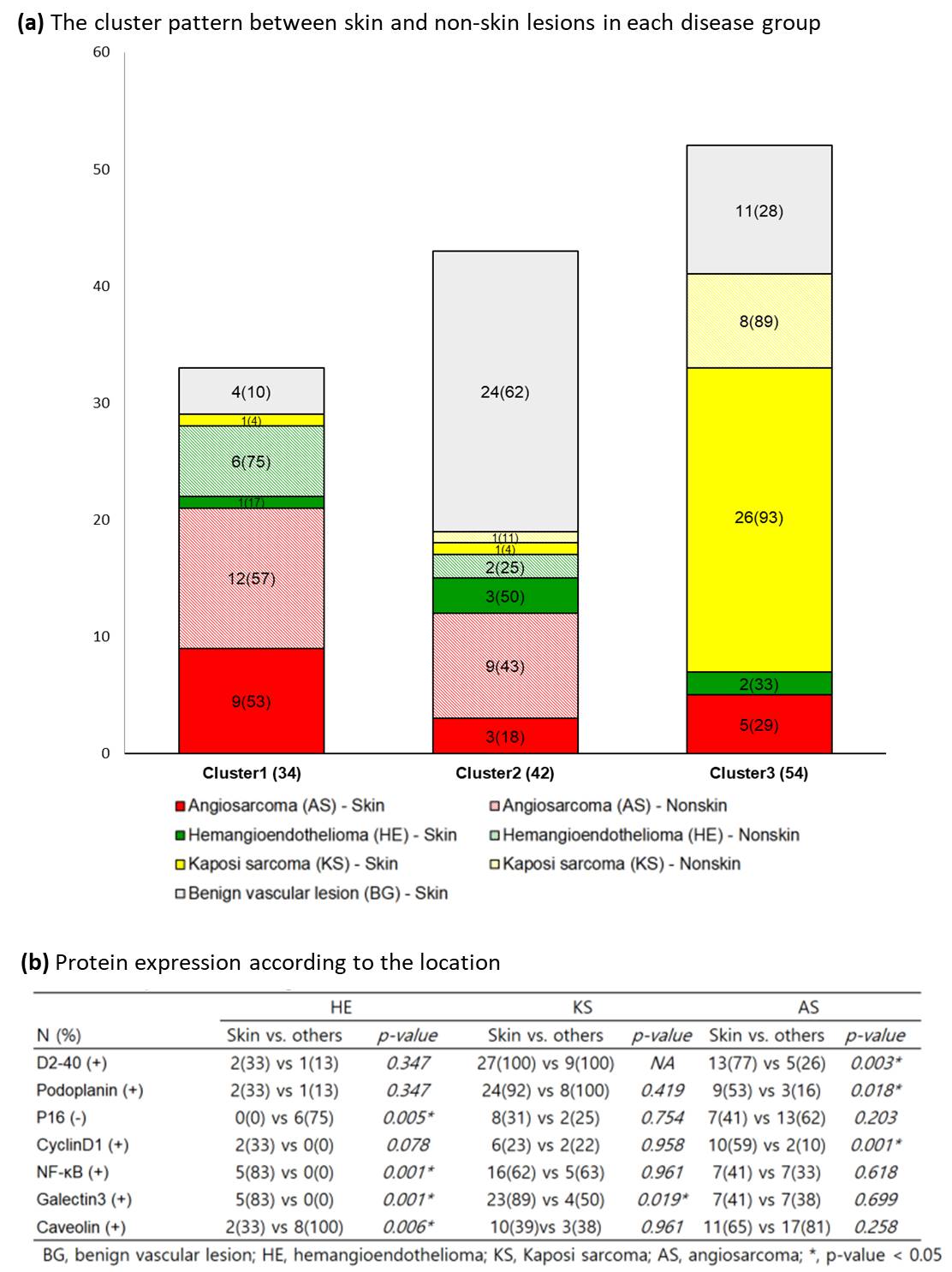

Supplement: Supplementary file 1 [file ijms-20-03142-s001.zip › ijms-515991-supplementary.jpg]
